# Significant Correlation Between Retinal Blood Flow and Oxygen Saturation During Intravitreal Aflibercept Treatment for Central Retinal Vein Occlusion

**DOI:** 10.1167/tvst.14.6.35

**Published:** 2025-06-30

**Authors:** Yukiko Miyoshi, Yuki Nakano, Yuta Koyama, Rie Osaka, Junichiro Akimitsu, Aki Booka, Kiyoshi Suzuma

**Affiliations:** 1 Department of Ophthalmology, Faculty of Medicine, Kagawa University, Miki-cho, Japan

**Keywords:** central retinal vein occlusion (CRV), OxymapT1, retinal venous oxygen saturation, laser speckle flowgraphy (LSFG), mean blur rate (MBR)

## Abstract

**Purpose:**

The purpose of this study was to evaluate the relationship between retinal blood flow and oxygen saturation during intravitreal aflibercept treatment for central retinal vein occlusion (CRVO) using OxymapT1 and laser speckle flowgraphy (LSFG).

**Methods:**

Thirty-two untreated patients (32 eyes) with nonischemic CRVO received monthly intravitreal aflibercept injections for 3 months followed by a pro re nata regimen; they were followed up for approximately 1 year. Central retinal thickness, retinal oxygen saturation, and retinal blood flow were measured using optical coherence tomography, OxymapT1, and LSFG, respectively.

**Results:**

Visual acuity (VA) and central retinal thickness (CRT; µm) significantly improved from 1 month onward in both the all-patient and nonischemic groups (VA baseline = 0.51, 0.48, 1 month = 0.30, 0.29, *P* = 0.049, *P* = 0.032; and CRT baseline = 615.0, 615.0, 1 month = 278.4, 275.0, *P* = 0.049, *P* < 0.001). Using OxymapT1, retinal venous oxygen saturation was reduced at baseline but significantly increased from 1 month after aflibercept injection and remained elevated in both groups (baseline = 34.1%, and 1 month = 41.1%, *P* = 0.006). Mean blur rate (MBR) remained stable overall, with a significant increase at the final visit in the nonischemic group (baseline = 22.4, and final = 27.4, *P* = 0.001). A significant positive correlation was found between venous oxygen saturation and MBR from 1 month after treatment onward (1 month = *R* = 0.538, *P* = 0.002).

**Conclusions:**

In CRVO, venous oxygen saturation and MBR were significantly correlated.

**Translational Relevance:**

Measuring and evaluating retinal blood flow and oxygen saturation during treatment of CRVO eyes is recommended.

## Introduction

Central retinal vein occlusion (CRVO) significantly affects retinal circulation, with findings of a global epidemiological study indicating a population of approximately 2.5 million patients with CRVO.^1^ In patients with CRVO, reduced retinal blood flow causes the body to produce vascular endothelial growth factor (VEGF),^2^ resulting in increased vascular permeability and macular edema.^3^ Approximately 80% of CRVO cases are nonischemic; however, in 34% of these cases, a shift to ischemic occurs during the course of the disease, resulting in a poor prognosis.^4^ Thus, understanding the dynamics of retinal circulation in patients with CRVO is crucial.

Ocular blood flow measurements are useful for a variety of ophthalmic diseases. Color Doppler imaging,^5^ laser Doppler velocimetry,^6^^,^^7^ laser speckle flowgraphy (LSFG; LSFG-NAVI; Softcare Co., Ltd., Fukuoka, Japan),^6^^,^^8^^–^^14^ and the retinal functional imager methodologies^13^ are all used as noninvasive techniques to evaluate ocular blood flow.

LSFG is a device that provides real-time imaging and display of the velocity distribution of scattered particles in the fundus, or fundus blood flow distribution; and it is able to measure retinal blood flow using the mean blur rate (MBR) of selected vascular regions. Studies of LSFG in CRVO have shown that, after anti-VEGF therapy, patients with an increased MBR had a better prognosis, but those without an increased MBR had a poorer prognosis.^15^^,^^16^ With CRVO, total capillary resistance increases and MBR decreases.^17^ In addition, following a 1-year follow-up after anti-VEGF treatment, researchers reported that MBR in the nonischemic group increased significantly after treatment, whereas MBR in the ischemic group did not increase.^18^

OxymapT1 is an instrument that can measure retinal oxygen saturation.^19^ Studies using OxymapT1 have revealed a lower venous oxygen saturation (VSO_2_) in eyes with CRVO compared with healthy eyes,^20^ and higher VSO_2_ after vitreous ranibizumab injection.^21^ Correlations between visual acuity and retinal large vessel oxygen saturation in patients with CRVO have also been reported.^22^^,^^23^

Although CRVO circulation has been evaluated in many studies, none have simultaneously used OxymapT1 and LSFG measurements to develop an understanding of the circulation dynamics of CRVO. Therefore, by simultaneously analyzing the results of both types of examinations, we aimed to elucidate the pathogenesis of CRVO from a previously unknown perspective.

## Methods

### Patients

The Kagawa University Faculty of Medicine Ethics Committee approved this retrospective study (approval number: H26-035). The patients provided written informed consent to participate, and the study followed the tenets of the Declaration of Helsinki.

This study included 32 eyes of 32 patients who visited the Department of Ophthalmology, Kagawa University Hospital, between May 2019 and August 2024. The patients were diagnosed with untreated nonischemic CRVO accompanied by macular edema. Treatment consisted of 3 intravitreal injections of aflibercept administered once a month, followed by pro re nata injections for a follow-up period of approximately 1 year.

The following measurements of patients were obtained at the initial visit and at the 1-, 2-, and 3-month follow-up visits and final visit within 1 year; visual acuity, central retinal thickness (CRT), measured using optical coherence tomography, retinal oxygen saturation measured using OxymapT1, and retinal blood flow measured using LSFG. Patients also underwent a fluorescein angiography examination at the initial visit. The Central Vein Occlusion Study Group defined an ischemic type as a case with more than 10 disc areas of nonperfusion.^24^ Based on this definition, we classified our entire cohort of patients (all group) as having nonischemic or ischemic CRVO (the nonischemic and converted groups, respectively).

Patients with a history of previous injections, photocoagulation, or vitrectomy and those who could not be accurately evaluated using imaging due to vitreous hemorrhage or strong lens opacity were excluded. Patients with ischemic CRVO were also excluded.

### Retinal Oximetry

Oximetry was performed using the OxymapT1 equipment (model T1; Oxymap, Reykjavik, Iceland),^20^^,^^21^^,^^25^^,^^26^ which uses a mydriatic fundus camera (Topcon TRC-50DX; Topcon Corporation, Tokyo, Japan). Normal fundus cameras have a digital camera attached to them to obtain fundus images. However, the OxymapT1 is attached to the digital camera. After the shutter releases, two monochromatic fundus images (570 nm and 600 nm) are captured using a built-in beam splitter, light filter, and control unit; the difference in their absorbance results in a visual color-coded display of oxygen saturation. Oxygen saturation was analyzed using the Oxymap Analyzer software. The images with an image quality graded < 6.0 were excluded.^25^^,^^27^^,^^28^

The vessels selected were measured within an area 1.5 to 3.0 times the diameter of the optic nerve papilla. All vessels whose progress could be followed were selected and analyzed. The same vessels were used throughout the process.

### LSFG Blood Flow Measurements

Measurements were performed using the LSFG-NAVI system (Softcare Co. Ltd., Fukuoka, Japan). This device visualizes and displays the real-time velocity distribution of scattered particles in the ocular fundus or the fundus blood flow distribution.^11^^,^^14^^,^^29^^–^^31^

We evaluated the MBR of the large vessels at the optic nerve head, as previously reported.^16^^–^^18^ A rubber band (analysis area) was set as an ellipse along the inner edge of the optic nerve papilla, and the vascular area was selected using software. The mean background tissue area was subtracted from the mean vascular area to obtain the MBR of the papillary large vessels. The mean MBR of the large vessels of the optic papilla was used in this study because it reflected the circulation of the entire retina.^16^^–^^18^

### Statistical Analysis

A repeated measures analysis of variance was performed on the following data items: arterial oxygen saturation (ASO_2_), VSO_2_, and arteriovenous oxygen saturation difference (ΔSO_2_) for the all group; VSO_2_ and ΔSO_2_ for the nonischemic group; and the logarithm of the minimum angle of resolution visual acuity (logMAR VA), CRT, ASO_2_, and VSO_2_ for the converted group. Friedman's test was conducted to analyze logMAR VA, CRT, and MBR for both the all and the nonischemic groups; ASO_2_ for the nonischemic group; and MBR and ΔSO_2_ for the converted group. Spearman’s rank correlation coefficient was used to determine the relationship between VSO_2_ and MBR.

All statistical analyses were performed using IBM SPSS Statistics for Windows version 28.0.1.0 (142; IBM Corp., Armonk, NY, USA).

The results are expressed as mean ± standard deviation. Statistical significance was set at *P* < 0.05.

## Results

This study included 32 untreated eyes from patients diagnosed with nonischemic CRVO at the initial visit using fluorescein fundus angiography. Of these, 6 patients progressed to ischemic CRVO within 3 to 12 months. Table 1 presents the clinical characteristics. The mean age was 70.9 ± 12.3 years for all patients, 69.8 ± 13.2 years in the nonischemic group, and 75.7 ± 4.1 years in the converted group. The average time from symptom onset to diagnosis was approximately 1.1 months across all groups. The mean follow-up duration was 10.3 ± 2.8 months overall. Comorbid conditions included hypertension (19 patients), diabetes (5), cerebral infarction (5), and cardiovascular disease (1). In the converted group, five patients had hypertension, one had cerebral infarction, and none had diabetes or cardiovascular disease.

**Table 1.** Clinical Characteristics of the Eyes With Central Retinal Vein Occlusion

Characteristic	All Group	Nonischemic Group	Converted Group
Number (male/female)	32 (19/13)	26 (17/9)	6 (2/4)
Age, years ± SD	70.9 ± 12.3	69.8 ± 13.2	75.7 ± 4.1
Time from CRVO onset to first visit, months ± SD	1.10 ± 1.30	1.10 ± 1.30	1.40 ± 1.60
Follow up period, months ± SD	10.3 ± 2.8	9.9 ± 2.9	12.0 ± 0.6
History, *n* (%)			
Hypertension	19 (59.4)	14 (53.8)	5 (83.3)
Diabetic mellitus	5 (15.6)	5 (19.2)	0 (0.0)
Cerebral infarction	5 (15.6)	4 (15.3)	1 (16.7)
Cardiovascular disease	1 (3.1)	0 (0)	1 (16.7)

CRVO, central retinal vein occlusion; SD, standard deviation.

All group, entire cohort; Converted group, patients with disease conversion to ischemic central retinal vein occlusion; Nonischemic group, patients with central retinal vein occlusion without ischemia.

**Figure 1.**
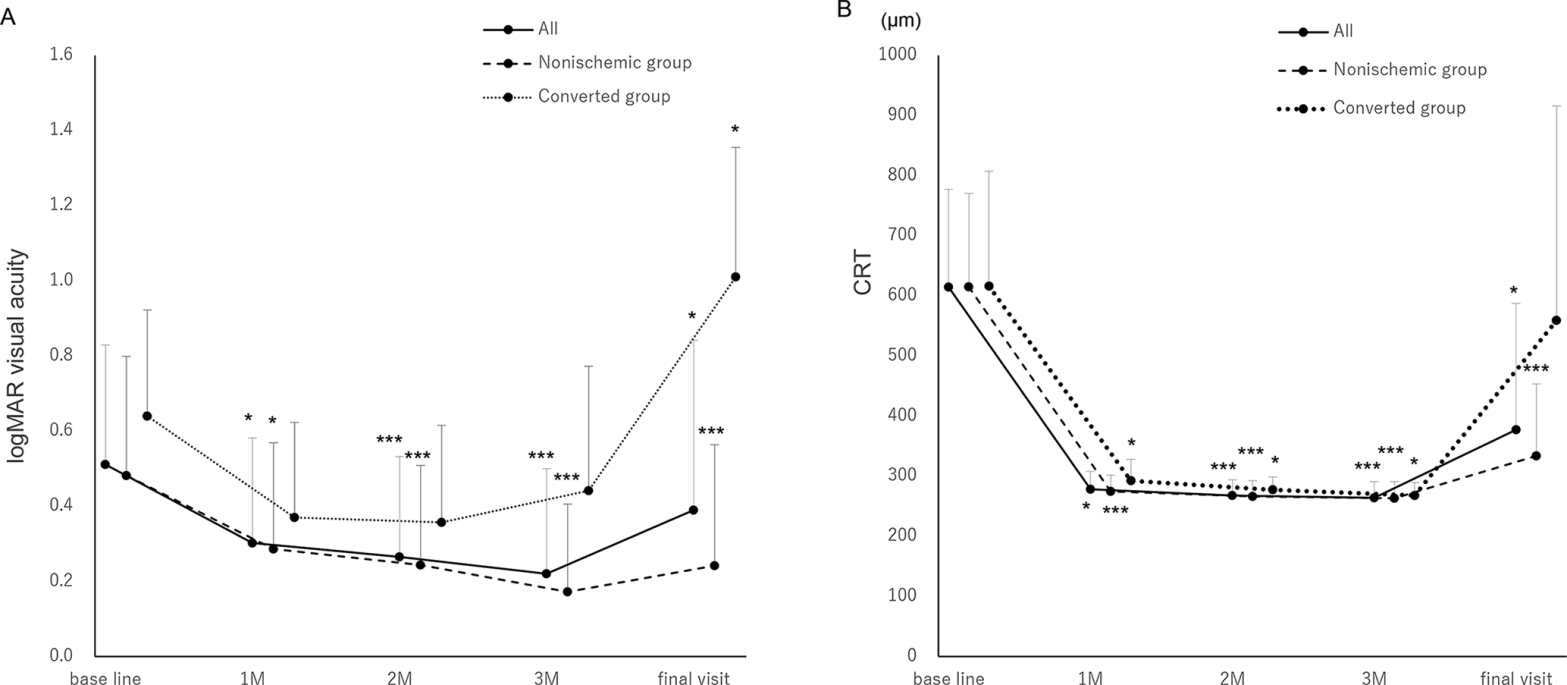
Changes in the mean logarithm of the minimum angle of resolution visual acuity and central foveal retinal thickness for each group. (**A**) Visual acuity is significantly improved in the all and nonischemic groups (1 month = *P* = 0.049 and *P* = 0.032; 2 months = *P* < 0.001 and *P* < 0.001; 3 months = *P* < 0.001 and *P* < 0.001; and final visit = *P* = 0.011 and *P* < 0.001, respectively; Freidman's test). However, in the converted group, visual acuity is significantly worsened at the final visit (final visit = *P* = 0.021; repeated measures analysis of variance). (**B**) Mean central foveal retinal thickness is significantly improved in the all and nonischemic groups (1 month = *P =* 0.049 and *P <* 0.001; 2 months = *P <* 0.001 and *P <* 0.001; 3 months = *P <* 0.001 and *P <* 0.001; and the final visit = *P =* 0.011 and *P* < 0.001, respectively; Freidman's test). Improvement in the mean CRT was maintained in the converted group; however, CRT increased and was not significant at the final visit (1 month = *P =* 0.042; 2 months = *P =* 0.031; 3 months = *P =* 0.026; and the final visit = *P =* 0.981; repeated measures analysis of variance). *P <0.05; **P <0.01; ***P <0.001. The all group includes the entire cohort; the converted group includes the patients with disease conversion to ischemic central retinal vein occlusion; the nonischemic group includes the patients with central retinal vein occlusion without ischemia. CRT, central retinal thickness; logMAR, logarithm of the minimum angle of resolution; M, month.

Figure 1A and Table 2 show the mean logMAR visual acuity changes for each group. The all and nonischemic groups showed significant improvement in logMAR VA (Freidman's test). The logMAR VA for the converted group showed no significant changes at 3 months, but significant worsening at the final visit (repeated measures analysis of variance).

Figure 1B and Table 3 show the changes in mean CRT. Mean CRT improved significantly in the all and nonischemic groups (Friedman's test). Improvement in the mean CRT was maintained in the converted group; however, a nonsignificant increase in CRT was observed at the final visit (repeated measures analysis of variance).

**Figure 2.**
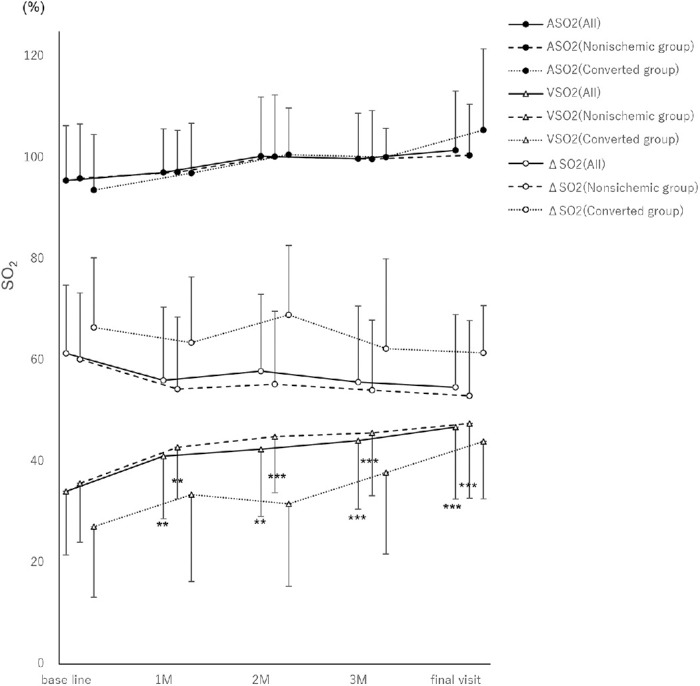
Changes in mean oxygen saturation. No significant changes in arterial oxygen saturation are observed in the all, nonischemic, and converted groups. No significant changes in retinal arteriovenous oxygen saturation differences are observed in the all, nonischemic, and converted groups. In the all and nonischemic groups, retinal venous oxygen saturation gradually and significantly increases (1 month = *P =* 0.006 and *P =* 0.007; 2 months = *P =* 0.001 and *P <* 0.001; 3 months = *P <* 0.001 and *P <* 0.001; and the final visit = *P <* 0.001 and *P <* 0.001, respectively). Venous oxygen saturation also increased gradually in the converted group, but not significantly (1 month = *P =* 0.813; 2 months = *P =* 0.937; 3 months = *P =* 0.389; and the final visit = *P =* 0.063; repeated measures analysis of variance). *P <0.05; **P <0.01; ***P <0.001. ASO_2_, arterial oxygen saturation; M, month; SO_2_, oxygen saturation; VSO_2_, venous oxygen saturation; ΔSO_2_, arterial oxygen saturation − venous oxygen saturation.

Figure 2 and Table 4 show the changes in mean oxygen saturation. The all, nonischemic, and converted groups showed no significant changes in ASO_2_. Similarly, no significant differences were observed in changes in ΔSO_2_ among the all, nonischemic, and converted groups. In the all and nonischemic groups, VSO_2_ trended upward and was significantly elevated at all time points. VSO_2_ also increased gradually in the converted group, but not significantly.

**Figure 3.**
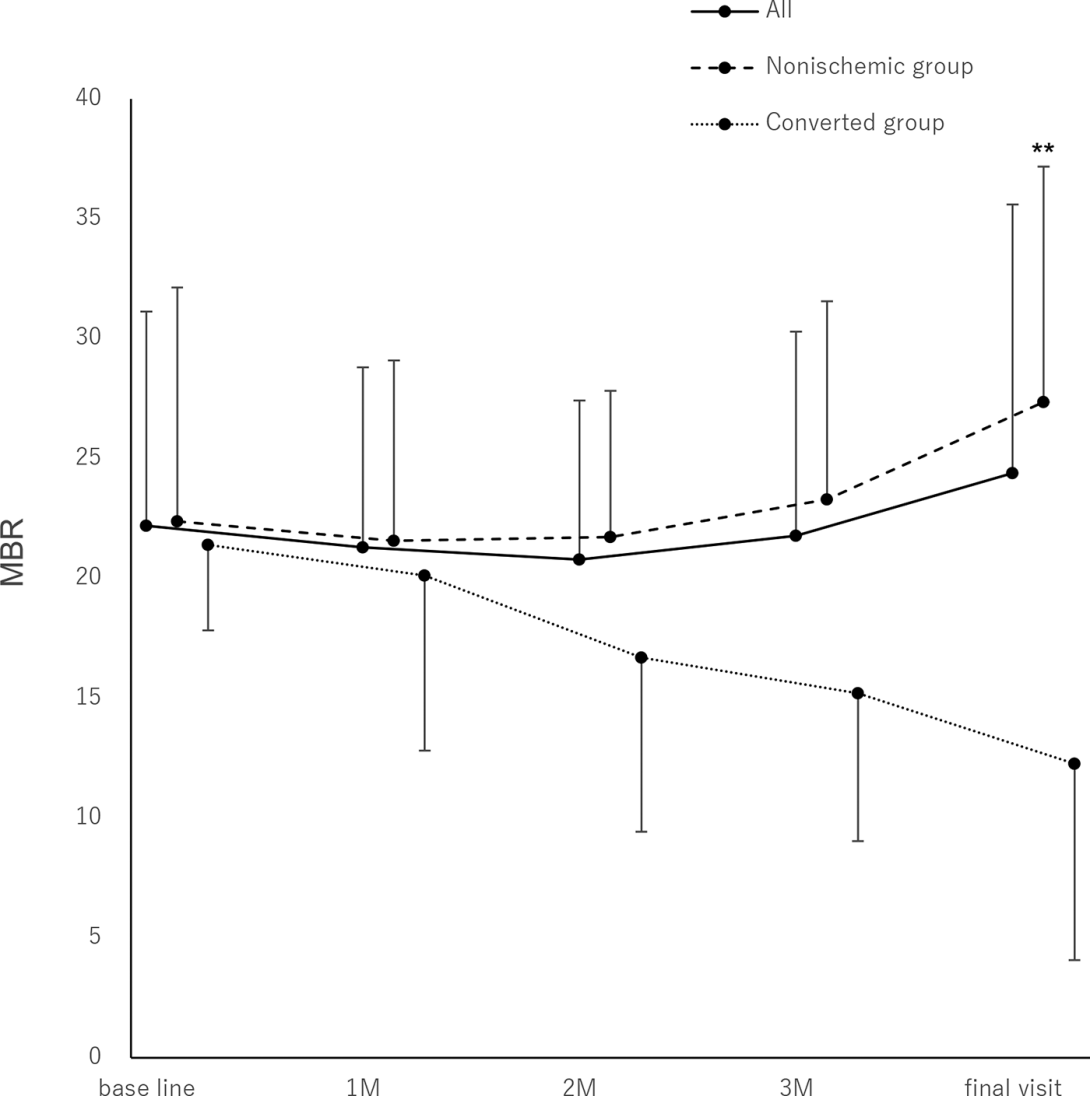
Changes in mean blur rate. No significant changes are observed in the all group. In the nonischemic group, there was a gradual increase and a significant increase at the final visit (final visit; *P* = 0.001) A gradual decreasing trend is observed in the converted group. all group, entire cohort; converted group, patients with disease conversion to ischemic central retinal vein occlusion; nonischemic group, patients with central retinal vein occlusion without ischemia. *P < 0.05; **P < 0.01; ***P < 0.001. M, month; MBR, mean blur rate.

Figure 3 and Table 5 show the changes in the mean MBR. The mean MBR did not change significantly in the all group. In the nonischemic group, the mean MBR was significantly increased at the final visit. A gradual decreasing trend, described previously,^18^ was observed in the converted group.

**Figure 4.**
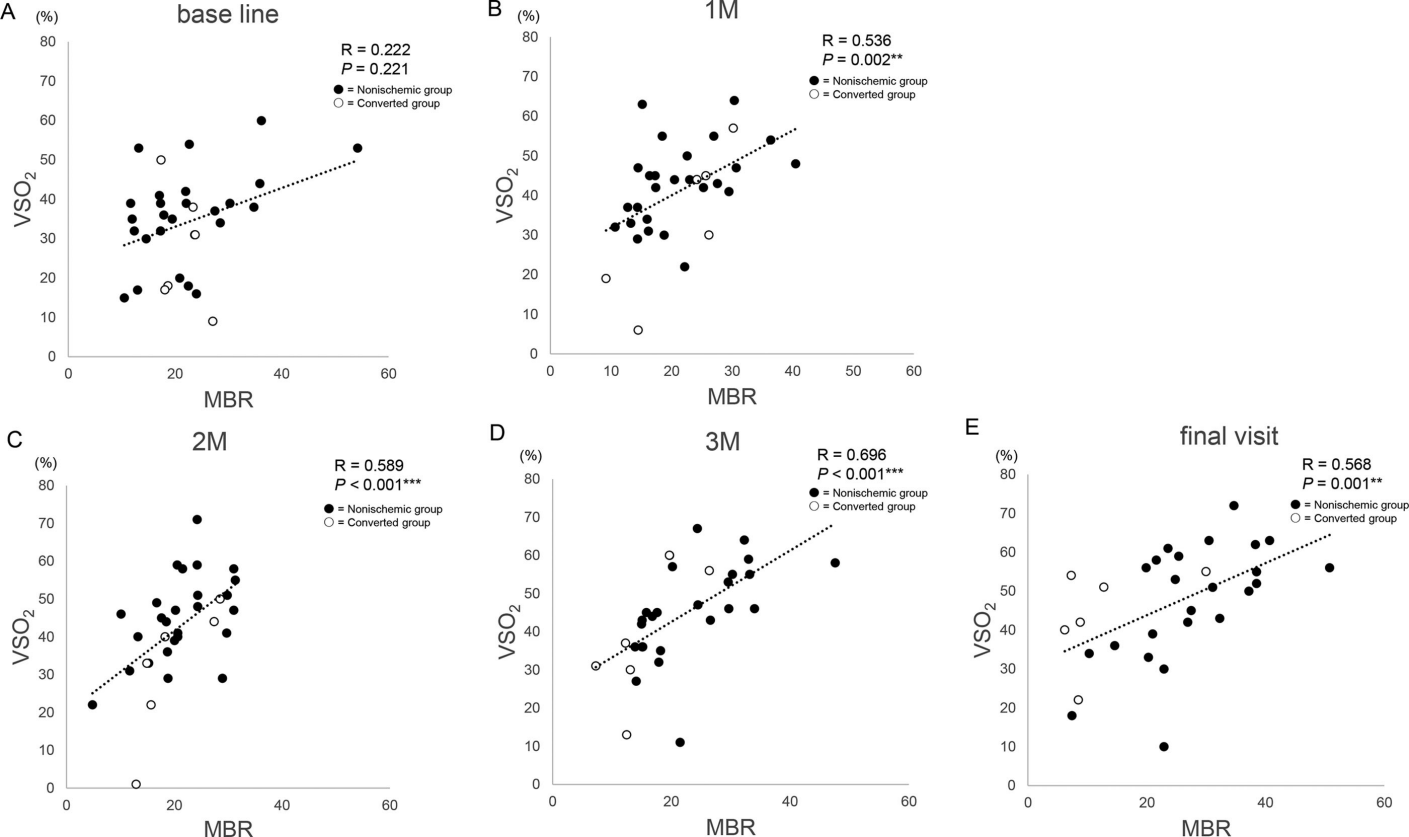
Scatter plots of venous oxygen saturation and mean blur rate. A positive correlation is shown between venous oxygen saturation and mean blur rate after anti-vascular endothelial growth factor therapy at (**A**) baseline = *R* = 0.571, *P =* 0.009; (**B**) 1 month = *R* = 0.672, *P =* 0.001; (**C**) 2 months = *R* = 0.772, *P <* 0.001; and (**D**) 3 months = *R* = 0.828, *P <* 0.001; and (**E**) the final visit = *R* = 0.568, *P <* 0.001. Spearman's correlation coefficient was used to determine the relationship between venous oxygen saturation and mean blur rate. *P < 0.05; **P < 0.01; ***P < 0.001. The all group includes the entire cohort; the converted group includes patients with disease conversion to ischemic central retinal vein occlusion; the nonischemic group indicates patients with central retinal vein occlusion without ischemia. M, month; MBR, mean blur rate; VSO_2_, venous oxygen saturation.

Figure 4 shows scatter plots of VSO_2_ the and MBR at (A) baseline, (B) 1 month after treatment, (C) 2 months after treatment, (D) 3 months after treatment, and (E) the final visit. A positive correlation was observed between VSO_2_ and MBR at (B) 1 month (*R* = 0.536, *P* = 0.002), (C) 2 months (*R* = 0.589, *P* < 0.001), (D) 3 months after treatment (*R* = 0.696, *P* < 0.001), and (E) the final visit (*R* = 0.568, *P* = 0.001; Spearman’s rank correlation coefficient).

Table 6 shows the changes in the mean blood pressure, intraocular pressure, and ocular perfusion pressure over the treatment and follow-up period. No significant differences were found at any time point.

**Table 2.** Changes in Mean logMAR Visual Acuity

	Baseline	1 Mo	2 Mo	3 Mo	Final Visit
All group	0.51 ± 0.32	0.30 ± 0.28	0.27 ± 0.27	0.22 ± 0.28	0.39 ± 0.45
		*P* = 0.049^*^	*P* < 0.001^***^	*P* < 0.001^***^	*P* = 0.011^*^
Nonischemic group	0.48 ± 0.32	0.29 ± 0.28	0.24 ± 0.27	0.17 ± 0.23	0.24 ± 0.32
		*P* = 0.032^*^	*P* < 0.001^***^	*P* < 0.001^***^	*P* < 0.001^***^
Converted group	0.64 ± 0.28	0.37 ± 0.25	0.36 ± 0.26	0.44 ± 0.33	101 ± 0.35
		*P* = 0.134	*P* = 0.106	*P* = 0.387	*P* = 0.021^*^

* *P* value < 0.05.

*** *P* value < 0.001.

**Table 3.** Changes in Mean CRT

	Baseline	1 Mo	2 Mo	3 Mo	Final Visit
All group, µm	615.0 ± 162.8	278.4 ± 29.3	268.0 ± 25.8	264.2 ± 26.7	377.4 ± 210.3
		*P* = 0.049^*^	*P* < 0.001^***^	*P* < 0.001^***^	*P* = 0.011^*^
Nonischemic group, µm	615.0 ± 155.2	275.0 ± 26.6	266.3 ± 26.6	263.5 ± 27.8	333.8 ± 119.9
		*P* < 0.001^***^	*P* < 0.001^***^	*P* < 0.001^***^	*P* < 0.001^***^
Converted group, µm	616.3 ± 191.5	292.5 ± 35.2	277.5 ± 21.1	268.0 ± 21.5	559.2 ± 357.2
		*P* = 0.042^*^	*P* = 0.031^*^	*P* = 0.026^*^	*P* = 0.981

* *P* value < 0.05.

*** *P* value < 0.001.

**Table 4.** Changes in Mean Oxygen Saturation

	Baseline	1 Mo	2 Mo	3 Mo	Final Visit
ASO_2_				
All group, %	95.5 ± 10.8	97.2 ± 8.6	100.3 ± 11.7	99.9 ± 9.0	101.5 ± 11.7
		*P* = 0.942	*P* = 0.170	*P* = 0.268	*P* = 0.052
Nonischemic group, %	96.0 ± 10.8	97.2 ± 8.3	100.3 ± 12.2	99.8 ± 9.6	100.5 ± 10.1
		*P* = 0.225	*P* = 0.709	*P* = 0.780	*P* = 0.576
Converted group, %	93.7 ± 11.0	97.0 ± 9.8	100.7 ± 9.2	100.2 ± 5.7	105.5 ± 16.1
		*P* = 0.979	*P* = 0.758	*P* = 0.803	*P* = 0.298
ΔSO_2_				
All group, %	61.4 ± 13.5	56.1 ± 14.5	57.9 ± 15.2	55.7 ± 15.0	54.7 ± 14.4
		*P* = 0.207	*P* = 0.616	*P* = 0.161	*P* = 0.067
Nonischemic group, %	60.2 ± 13.1	54.3 ± 14.3	55.3 ± 14.4	54.1 ± 13.9	53.0 ± 14.9
		*P* = 0.184	*P* = 0.351	*P* = 0.162	*P* = 0.067
Converted group, %	66.5 ± 13.8	63.5 ± 13.0	69.0 ± 13.7	62.3 ± 17.7	61.5 ± 9.3
		*P* = 1.000	*P* = 1.000	*P* = 1.000	*P* = 1.000
VSO_2_				
All group, %	34.1 ± 12.6	41.1 ± 12.4	42.5 ± 13.3	44.2 ± 13.6	46.8 ± 14.2
		*P* = 0.006^**^	*P* = 0.001^**^	*P* < 0.001^***^	*P* < 0.001^***^
Nonischemic group, %	35.7 ± 11.6	42.8 ± 10.2	45.0 ± 11.1	45.7 ± 12.4	47.5 ± 14.8
		*P* = 0.007^**^	*P* < 0.001^***^	*P* < 0.001^***^	*P* < 0.001^***^
Converted group, %	27.2 ± 14.0	33.5 ± 17.2	31.7 ± 16.3	37.8 ± 16.1	44.0 ± 11.4
		*P* = 0.813	*P* = 0.937	*P* = 0.389	*P* = 0.063

** *P* value < 0.01.

*** *P* value < 0.001.

**Table 5.** Changes in Mean MBR

	Baseline	1 Mo	2 Mo	3 Mo	Final Visit
All group	22.2 ± 8.9	21.3 ± 7.5	20.8 ± 6.6	21.8 ± 8.5	24.4 ± 11.2
		*P* = 1.000	*P* = 1.000	*P* = 1.000	*P* = 0.647
Nonischemic group	22.4 ± 9.8	21.6 ± 7.5	21.7 ± 6.1	23.3 ± 8.3	27.4 ± 9.8
		*P* = 0.929	*P* = 0.623	*P* = 0.195	*P* = 0.001^**^
Converted group	21.4 ± 3.6	20.1 ± 7.3	16.7 ± 7.3	15.2 ± 6.2	12.3 ± 8.2
		*P* = 1.000	*P* = 0.679	*P* = 0.285	*P* = 0.176

** *P* value < 0.01.

**Table 6.** Changes in Mean Blood Pressure, Intraocular Pressure, and Ocular Perfusion Pressure Over the Treatment and Follow-Up Period

Characteristic	Baseline	1 Mo	2 Mo	3 Mo	Final Visit
Mean blood pressure, mm Hg	100.6 ± 15.8	96.2 ± 14.8	94.9 ± 13.4	94.0 ± 17.1	94.4 ± 15.1
		*P* = 0.317	*P* = 0.101	*P* = 0.035^*^	*P* = 0.063
Intraocular pressure, mm Hg	14.6 ± 2.8	14.2 ± 2.9	14.3 ± 3.2	14.3 ± 3.2	15.8 ± 4.1
		*P* = 0.968	*P* = 0.978	*P* = 0.974	*P* = 0.147
Ocular perfusion pressure, mm Hg	52.5 ± 10.3	49.9 ± 10.1	48.9 ± 9.0	48.4 ± 11.3	48.8 ± 10.3
		*P* = 0.777	*P* = 0.409	*P* = 0.163	*P* = 0.199

* *P* value < 0.05.

## Discussion

This is the first study to simultaneously measure retinal oxygen saturation with OxymapT1 and retinal blood flow with LSFG before and after anti-VEGF therapy for CRVO.

The Central Retinal Vein Occlusion Study Group reported that the percentages of cases of CRVO that transitioned from the nonischemic to the ischemic type at 4 months and 3 years were 15% and 34%, respectively.^32^ In the present study, 6 of the 32 patients developed ischemic CRVO 3 months to 1 year after the initial visit, which is consistent with the findings of previous studies. In one study, the mean age of patients with nonischemic CRVO was 69.8 ± 13.2 years, whereas that of patients who transitioned to the ischemic type was 75.7 ± 4.1 years.^32^ All the patients in the present study were slightly older (69.1 ± 12.9 years), and those with disease that transitioned to the ischemic form were aged 78.0 ± 2.5 years. Consistent with the results of the GALILEO,^33^ and COPERMICUS^34^ studies, visual acuity improved significantly and the CRT decreased after intravitreal injection of aflibercept.

Studies using OxymapT1 have shown that retinal ASO_2_ is unaffected in eyes with CRVO, whereas retinal VSO_2_ is reduced.^20^ This is considered to be due to venous occlusion, which reduces retinal blood flow and oxygen supply to the tissue. Hypoxic tissues consume more oxygen from the volume of blood per unit area because the volume of blood passing through the capillary bed is reduced. This is why VSO_2_ is thought to decrease in eyes with CRVO.^20^ Similarly, in eyes with CRVO, retinal VSO_2_ is reduced because venous occlusion lowers the blood flow, thus prolonging the transient time, and resulting in the ischemic retina consuming more oxygen.^35^ The oxygen saturation in retinal veins increases after a vitreous injection of ranibizumab; this is considered to be due to the formation of an arteriovenous shunt.^21^ We found a similar pattern in our results: the retinal VSO_2_ decreased in eyes with CRVO and increased after aflibercept treatment.

Through measurements obtained with LSFG, the MBR was shown to increase after a vitreous injection of bevacizumab in eyes with CRVO.^16^ In the present study, the MBR was maintained in the nonischemic group after vitreous injection and significantly increased at the final visit. The reason why the difference was significant only in the final visit may be because patients with relatively severe disease and older ages were included in our study.

The MBR tended to decrease after vitreous injection in the converted group, although not significantly. Patients in the converted group underwent pan retinal photocoagulation (PRP) after conversion to ischemic CRVO. The reason for the decrease in MBR may be that the condition worsened and blood flow was further slowed down. Another possible reason is PRP was performed before the final visit: the blood flow was reported to decrease after PRP in diabetic retinopathy.^36^

In a recent study, retinal oxygen saturation was measured simultaneously with blood flow using OxymapT1 and Doppler optical coherence tomography, respectively, in healthy persons.^37^ Such simultaneous measurement has not been reported in patients with CRVO. Some studies have examined the MBR or VSO_2_, individually, in patients with CRVO; however, the present study is the first to examine the relationship between oxygen saturation and blood flow in CRVO.

Surprisingly, in the present study, we found strong positive correlations between VSO_2_ and MBR after anti-VEGF therapy. Our results showed that VSO_2_ decreased with decreasing blood flow, possibly suggesting that oxygen diffusion increases and saturation decreases when blood flow slows.

Retinal hypoxia has been observed in diabetic retinopathy. Arteriovenous oxygen differentials decrease with increasing severity of diabetic retinopathy; assuming that this is due to capillary dropout and nonperfusion, leading to decreased oxygen supply to the tissues, is reasonable.^38^

In healthy eyes, the relationship between the MBR and ocular perfusion pressure is bilinear within a certain range.^39^^,^^40^ Therefore, to rule out a physiological response, ocular perfusion pressure was calculated. However, based on the results of the present study, we considered that ocular perfusion pressure was not associated with the MBR because no significant changes were observed.

In eyes with CRVO, occlusion of the central retinal vein reduces blood flow, causing retinal ischemia and hypoxia. The ischemia of the retina leads to production of VEGF^2^ and increased vascular resistance.^17^ Thus, a vicious cycle of CRVO is triggered. Therefore, we believe that measuring and evaluating retinal blood flow and oxygen saturation during treatment of CRVO eyes is essential.

### Limitations

This study was limited by the small number of cases included, the fact that all the participants were Asian, and its single-center design. Further studies are needed that include more cases.

Moreover, whereas the natural course of the disease may influence changes in retinal blood flow and oxygen saturation during follow-up, it is ethically infeasible to observe untreated patients over time. As such, this aspect could not be investigated in the present study.

PRP was administered exclusively to patients in the converted group, and all treatments were performed after the 3-month follow-up point. Therefore, it is possible that the effects of PRP influenced the findings at the final visit in both the all group and the converted group.
